# The overlooked connection: Managing menstrual irregularities in chronic conditions - A scoping review

**DOI:** 10.1177/17455057261493736

**Published:** 2026-09-29

**Authors:** Jennifer L. Dueck, Hilary Hwu, Simin Popat, Alena K. Thompson, Nicole L. Tegg, Colleen M. Norris

**Affiliations:** 1 Faculty of Nursing, 70410 University of Alberta, Edmonton, AB, Canada; 2 Cavarzan Chair in Women’s Research, WCHRI, Edmonton, AB, Canada; 3 Faculty of Medicine, School of Public Health Sciences, University of Alberta, Edmonton, AB, Canada

**Keywords:** menstrual irregularities, chronic condition, cardiovascular disease, women’s health, scoping review

## Abstract

**Background:**

Menstrual irregularities are frequently reported among women living with chronic conditions, yet menstrual health remains inconsistently assessed and poorly integrated into chronic condition management. The extent to which menstrual irregularities are reported, interpreted, and managed across chronic condition contexts has not been comprehensively synthesized.

**Objective:**

To map the existing evidence on the prevalence, types, and management of menstrual irregularities among women of reproductive age with chronic conditions.

**Eligibility criteria:**

Eligible studies included biological females aged 18–45 years with chronic conditions, including cardiovascular disease, chronic kidney disease, diabetes, inflammatory bowel disease, and liver disease, reporting menstrual irregularities.

**Sources of evidence:**

A scoping review was conducted in accordance with the Joanna Briggs Institute methodology and reported following PRISMA-ScR guidelines. Systematic searches of MEDLINE, CINAHL, Embase, Scopus, and Web of Science, along with grey literature sources, were conducted for English-language studies published from 2010 to 2025. Data was extracted and synthesized.

**Charting methods:**

Four independent reviewers used a standardized form to extract study design, population characteristics, chronic condition, menstrual irregularities, and key findings; discrepancies were resolved through consensus.

**Results:**

Twenty-six studies from 15 countries met the inclusion criteria. Across categories of chronic condition, the most reported menstrual irregularities included amenorrhea, oligomenorrhea, polymenorrhea, and menorrhagia, and were consistently associated with adverse cardiometabolic, inflammatory, and quality-of-life outcomes. Reported interventions were heterogeneous and largely symptom-focused, with limited attention to underlying systemic or hormonal mechanisms.

**Conclusions:**

Menstrual irregularities are common among women with chronic conditions and may serve as early clinical markers of systemic dysfunction and disease progression. Integrating standardized menstrual assessment into chronic condition management represents an important opportunity to improve early detection, risk stratification, and patient-centered care. Future research should prioritize longitudinal and interventional studies to inform evidence-based, multidisciplinary models that embed menstrual health as a core component of chronic condition care.

## Introduction

Chronic conditions, including cardiovascular disease (CVD), chronic kidney disease (CKD), and diabetes mellitus, are long-lasting health conditions that develop from a combination of genetic, physiological, environmental, and lifestyle factors.^1^ Women with CVD,^2–11^ CKD,^12–19^ diabetes mellitus,^20–25^ and gastrointestinal^26^ or liver disorders^27^ frequently experience menstrual irregularities. These can range from cycle irregularities to amenorrhea and arise from a combination of physiological, hormonal, and psychological factors associated with chronic disease. Alterations in the estrogen-progesterone feedback loop can disrupt normal hormone regulation and contribute to menstrual dysfunction.^28,29^ In addition, treatments and medications used to manage chronic conditions may further alter menstrual function.^28,29^

One mechanism through which chronic conditions may influence menstrual health is allostasis and the accumulation of allostatic load, which describe the body’s adaptive responses to persistent physiological stress.^30^ Chronic illness represents a sustained biological stressor that activates neuroendocrine, immune, metabolic, and autonomic regulatory systems, including the hypothalamic-pituitary-adrenal (HPA) axis.^30^ Although these adaptations are initially protective, prolonged activation results in cumulative allostatic load, leading to dysregulation of hypothalamic signaling and suppression of the hypothalamic-pituitary-gonadal (HPG) axis.^31^ Chronic HPA axis activation, together with inflammatory and metabolic signaling, disrupts the pulsatile secretion of gonadotropin-releasing hormone (GnRH), reducing downstream luteinizing hormone (LH) and follicle-stimulating hormone (FSH) secretion, impairing ovarian steroidogenesis and ovulation, and ultimately manifesting clinically as functional hypothalamic hypogonadism, menstrual irregularities, or secondary amenorrhea.^31,32^ This proposed mechanism may account for the consistent pattern of menstrual irregularities identified in this review across chronic conditions with otherwise distinct underlying pathophysiology.

Menstrual irregularities have important implications for overall health and chronic disease outcomes, beyond reproductive effects. Estrogen and progesterone play essential roles in maintaining vascular health by regulating blood flow, lipid metabolism, and endothelial function.^33,34^ When menstrual cycles become irregular, estrogen levels frequently decline,^20,31,32^ decreasing its cardioprotective effects and increasing vulnerability to cardiovascular dysfunction.^35^ Hormonal dysregulation associated with chronic conditions may also promote systemic inflammation, elevated blood pressure, and accelerated atherosclerosis,^35,36^ further increasing cardiovascular risk.^37,38^ Accordingly, menstrual irregularities during the reproductive years have been associated not only with an increased risk of cardiovascular disease^9,11,35,39^ but also with osteoporosis,^40^ neurological symptoms,^41^ cognitive dysfunction,^42^ and premature mortality.^43^ Importantly, the consequences of untreated or poorly managed menstrual dysfunction extend beyond reproductive health and are associated with substantial reductions in women’s quality of life.^26^ Given that chronic conditions affect more than one-third of Canadian women, these findings underscore the importance of timely identification and effective management of menstrual dysfunction.^44^

Despite the recognized association between chronic conditions and menstrual irregularities, there remains a significant gap in synthesized evidence examining this relationship. While research continues to explore the broader effects of chronic disease on women’s health, relatively few studies have specifically investigated its impact on menstrual health or evaluated strategies for identifying and managing menstrual dysfunction. Furthermore, the long-term health consequences of chronic condition-related menstrual irregularities remain poorly understood.^35,45^

This scoping review aimed to systematically examine the existing evidence on the prevalence, types, and management of menstrual irregularities among women of reproductive age living with chronic conditions. For the purposes of this review, we focused on literature pertaining to females (defined as persons with a uterus), recognizing that this construct is not interchangeable with the term *women*. However, throughout this review, we use the terminology reported in the original studies to accurately reflect the source literature.

## Methods

This scoping review was conducted per the JBI methodology for scoping reviews^46^ and reported in line with the Preferred Reporting Items for Systematic Reviews and Meta-Analyses extension for Scoping Reviews (PRISMA-ScR).^47^ An a priori protocol was followed, registered on the Open Science Framework (https://osf.io/e549b). As the objective of this review was to synthesize current knowledge and delineate gaps in the management and outcomes of menstrual irregularities among women with chronic conditions, a scoping review methodology was the most appropriate approach. This design allows for comprehensive mapping of evidence across both scholarly and grey literature. The PRISMA-ScR reporting checklist can be found in the supplemental file.

### Review questions

This scoping review aimed to address the following research questions:

1. What is the current scope of knowledge regarding menstrual irregularities in women with chronic conditions?
2. What are the reported management strategies for menstrual irregularities in chronic conditions?
3. What are the knowledge gaps in menstrual cycle management in chronic conditions?

### Inclusion criteria

#### Population

We considered studies that included biological females aged 18 to 45 years old, with an upper limit of 45 years to reflect early natural menopause.^48^ Studies involving participants outside the 18- to 45-year age range were included if age-stratified data were available.

### Concept

Studies that involved menstrual irregularities in females experiencing chronic conditions were included in this review. In the context of this review, menstrual irregularities were defined as secondary amenorrhea, oligomenorrhea, polymenorrhea, menorrhagia, hypomenorrhea, dysmenorrhea, and metrorrhagia.^49^ Secondary amenorrhea is defined as the temporary or permanent cessation of periods in someone who has experienced normal menstruation; oligomenorrhea as abnormally infrequent or scanty menstrual flow; polymenorrhea as menstruation at abnormally frequent intervals; menorrhagia as abnormally profuse menstrual flow; hypomenorrhea as decreased menstrual flow; dysmenorrhea as painful menstrual periods; and metrorrhagia as irregular uterine bleeding, especially between periods.^50^

Chronic conditions are long-lasting health conditions that develop from a combination of genetic, physiological, environmental, and lifestyle factors.^1^ This review included CVD, CKD, type 1 diabetes mellitus (T1DM), type 2 diabetes mellitus (T2DM), inflammatory bowel disease (IBD), and liver disease.

Studies focusing exclusively on menstrual irregularities related to hormonal causes, including primary ovarian insufficiency (POI) and polyendocrine metabolic ovarian syndrome (PMOS), eating disorders, thyroid disorders, hormonal birth control, intrauterine devices (IUDs), pregnancy, menopause, hyperprolactinemia, endometriosis, or uterine fibroids, were excluded. Additionally, studies examining menstrual irregularities amenable to surgical intervention were not considered.

### Context

Studies in all settings, including both healthcare and non-healthcare settings, were considered without restrictions on geographical location.

### Types of evidence sources

Types of evidence considered included quantitative, qualitative, and mixed-methods study designs, such as randomized controlled trials (RCTs), non-randomized controlled trials, prospective and retrospective cohort studies, case-control studies, systematic reviews, and grey literature.

### Search strategy

A two-phase search strategy to comprehensively identify published studies was used. This search occurred between May and August 2025. Phase One included an initial limited search of MEDLINE (via PubMed) and CINAHL (via EBSCOhost), conducted using preliminary keywords based on existing field knowledge. This step helped identify key terms and concepts. Secondly, we analyzed the titles and abstracts of relevant articles to extract text words and index terms used to describe relevant studies. The analysis informed the development of a comprehensive and database-specific search strategy for each included database.

Phase Two involved conducting a full search using the refined search strategies across multiple electronic databases, including *MEDLINE (via PubMed), CINAHL (via EBSCOhost), Embase, Scopus, and Web of Science.* The final search strategy incorporated controlled vocabulary and free-text keywords, applying Boolean operators (AND, OR), truncation, and proximity searching to maximize the retrieval of relevant literature. The key search terms were “menstrual irregularities,” “menstrual abnormalities,” “menstrual dysfunction,” “amenorrhea,” “chronic disease,” “chronic illness,” and “non-communicable disease,” along with condition-specific terms such as “cardiovascular disease,” “CVD,” “diabetes,” and “chronic kidney disease,” “type 1 diabetes,” “type 2 diabetes”, “inflammatory bowel disease,” “IBD,” “chronic kidney disease,” “CKD,” and “liver disease.”

A sample MEDLINE (via PubMed) search strategy included (“menstrual irregularities” OR “amenorrhea”) AND (“chronic disease” OR “noncommunicable disease”) AND (“hormone therapy” OR “clinical management” OR “lifestyle intervention”)*.* The search strategy was adapted for each database, and the final versions are documented in the supplemental file. To ensure comprehensive coverage, grey literature sources were also searched, including the World Health Organization (WHO), the Canadian Institute of Health Information (CIHI), and Health Canada.

Studies published from 2010 onwards were included because substantial advances in women’s health research, menopause care, and evidence-based clinical practice over the past decade mean that more recent studies are more representative of contemporary clinical management.^51^ The primary language of inclusion was English; therefore, we did not consider studies published in languages other than English. The full search strategies are provided in the supplemental file.

### Study/Source of evidence selection

Following the search, all identified citations were collated and uploaded to Covidence (Veritas Health Innovation, Melbourne, Australia)**,** where duplicates were removed. All screening stages were performed by two independent reviewers. A pilot test with 1.4% (50) titles and abstracts was performed (J.D. and S.P.) and yielded a Cohen’s κ of 0.41 (weak interrater agreement). The team met to ensure the inclusion and exclusion criteria were clearly defined and consistently understood by all members. The same two reviewers conducted a second pilot test on an additional 50 studies, yielding a Cohen’s κ of 0.85 (strong interrater agreement). Following calibration, the remaining titles, abstracts, and full-text articles were independently screened by reviewer pairs according to the standardized eligibility criteria. Full-text articles deemed potentially eligible were retrieved and assessed against the predefined inclusion and exclusion criteria. Additional inter-rater reliability statistics were not calculated, and any disagreements at any stage of the screening process were resolved through discussion or, when necessary, consultation with a third reviewer. The results of the search and study selection process are reported using a PRISMA-ScR Flow Diagram (Figure 1).^47^

**Figure 1.**
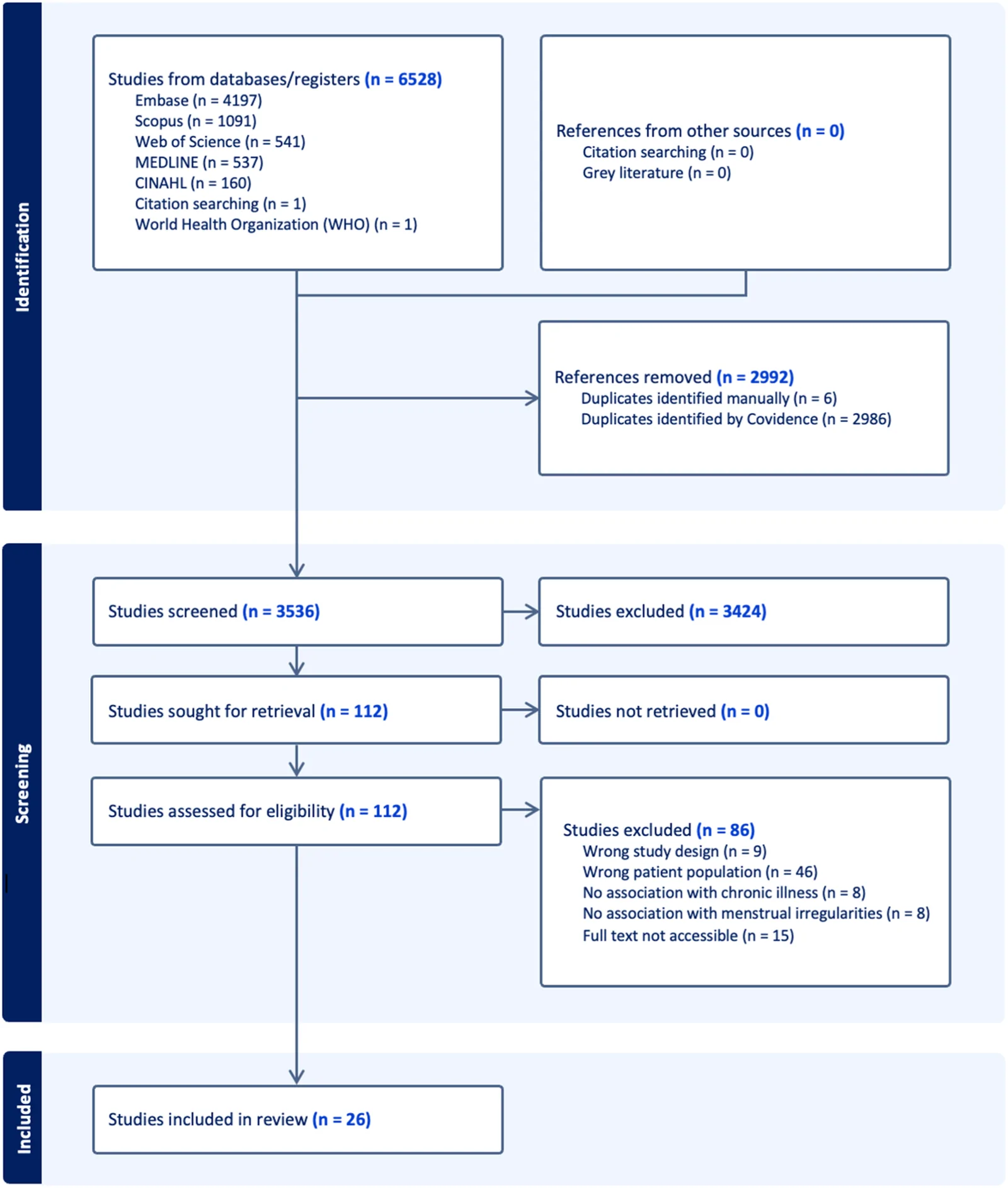
PRISMA-ScR Flow Diagram of Article Selection Process.

### Data extraction

Data were extracted using the standardized JBI data extraction instrument.^46^ A pilot test was done (J.D. and S.P.) with 10% of the studies to ensure sufficient data were consistently extracted and to verify congruence in extracted variables. Data extraction included details about the study characteristics (e.g., author(s), year of publication, study location, and study design), population characteristics (e.g., age, type of chronic condition, and type of menstrual irregularity), interventions or management strategies (e.g., hormonal therapy, pharmacological treatments, lifestyle modification, and clinical interventions) and results and key findings related to menstrual health (e.g., menstrual cycle regulation, estrogen levels, coronary vascular risks such as high blood pressure, lipid levels and atherosclerosis progression). Disagreements were resolved through discussion with an additional reviewer when necessary. Data extraction tool available in supplemental files.

### Data synthesis

The extracted data were analyzed to synthesize findings, identify patterns, trends, and gaps in the literature. Results are presented narratively alongside tabulated data. This summary describes how the findings align with the review objectives and highlights key themes, patterns, associations, and gaps in the literature. A structured approach was used to provide a comprehensive overview of existing evidence, identify gaps in clinical practice and research on the management of menstrual irregularities in women with chronic conditions, and outline future directions.

### Ethics approval and consent to participate

As this scoping review used anonymized data from previously published studies, this work does not require approval from an institutional review board or informed consent.

## Results

### Study inclusion

A total of 6528 titles were retrieved. After duplicates were removed, 3536 studies were screened for eligibility based on title and abstract. Of these, 112 full texts were retrieved and assessed; 86 articles were excluded. The remaining 26 articles were included.

### Characteristics of included studies

The 26 articles, published in 15 countries, were all published in English between 2010 and 2025. There were nine studies from the USA, three from China, two from Iran, and one each from Egypt, Australia, France, Finland, India, Denmark, the UK, South Korea, Japan, Canada, Poland, and Chile. Study designs were primarily observational.

Across the 26 included studies, menstrual irregularities were measured using heterogeneous approaches. The majority relied on participant self-report obtained through questionnaires or structured interviews, either prospectively or retrospectively. Several studies extracted menstrual information from electronic medical records or retrospective chart reviews, while only a limited number collected menstrual data prospectively as part of longitudinal cohort follow-up. No study employed standardized prospective menstrual diaries or validated menstrual tracking instruments across all participants. This heterogeneity in menstrual assessment should be considered when interpreting reported prevalence estimates and intervention outcomes. The characteristics of the included studies are reported in Table 1.

**Table 1.** Characteristics of Included Studies.

Study, Design & Country	Participant Characteristics	Chronic Condition	Menstrual Irregularity	Key findings	Method of menstrual cycle data collection
Chung et al., 2021^2^ Cohort Australia	N=7729 37-42 years	CVD	Menorrhagia, Oligomenorrhea, Polymenorrhea	● HTN in women was associated with more frequent painful, heavy, and irregular menstrual cycles, especially among those with additional risk factors (e.g., older age, higher BMI, stress, early menarche, rural residence, lower education, parity). ● Clinicians should consider more frequent blood pressure screening in young women with menstrual abnormalities, particularly heavy or irregular periods, given rising HTN rates.	● Self-reported by participants through structured survey questions about their experiences in the previous 12 months.
Dubey et al., 2023^4^ Cross-sectional United States	N=291 12-40 years	CVD	Menorrhagia	● Women hospitalized for menorrhagia had ∼2× higher odds of MACE (e.g., CHD, heart failure, MI), with stronger risk when cycles were regular. ● Supports routine screening and management of menorrhagia as part of cardiovascular disease prevention in women.	● Data collected using hospital discharge data and classified based on specific ICD-10 codes for heavy menstrual bleeding, with further distinction based on menstrual regularity.
Heliovaara-Peippo et al., 2011^51^ RCT Finland	N=236 35-49 years	CVD	Menorrhagia	● Hysterectomy for menorrhagia is linked to higher long-term inflammation and greater diabetes medication use, suggesting increased cardiovascular risk. ● Hysterectomy for menorrhagia may increase cardiovascular risk	● A combination of laboratory measurements (alkaline haemitin method), structured questionnaires at multiple time points, and review of medical records for treatment changes due to bleeding problems.
Iqbal & Vijaya, 2025^5^ Retrospective observational India	N=200 35-45 years	CVD	Menorrhagia, Polymenorrhea, Oligomenorrhea	● ∼50% of women with abnormal uterine bleeding (AUB) had comorbidities (HTN, diabetes, hypothyroidism). ● These conditions may contribute to AUB via vascular fragility and endothelial dysfunction.	● A structured, retrospective review of medical records, focusing on specific menstrual parameters and types of AUB, and quantified using descriptive statistics.
Khajali et al., 2016^37^ Cross-sectional study Iran	N=304 15-46	CVD	Amenorrhea, Polymenorrhea, Menorrhagia, Oligomenorrhea	● Women with congenital heart disease (especially complex/cyanotic) have delayed menarche and more menstrual abnormalities, particularly menorrhagia; amenorrhea occurs in complex cases. ● Highlights the need for ongoing gynecologic care to manage menstrual and reproductive health in this population.	● Self-reported by the participants during their clinic visits, using standardized definitions provided by the research team.
Lai et al., 2024^3^ Mendelian randomization China	N=455, 478 18-50 years	CVD	Menorrhagia, Polymenorrhea, Oligomenorrhea	● Menstrual disorders are linked to higher cardiovascular risk via metabolic and hormonal pathways (e.g., insulin resistance, androgen excess). ● Menstrual irregularities may serve as early markers for identifying women at increased CVD risk.	● Self-reported symptoms, which were subsequently documented in health records using ICD codes. ● Data are both self-reported and retrospective chart review.
Matsushita et al., 2022^39^ Cross-sectional study Japan	N=20 16-39 years	CVD	Menorrhagia	● ∼42% of women with Fontan circulation experience menorrhagia, linked to cardiac dysfunction and secondary erythrocytosis. ● Managing menstrual flow (e.g., with contraceptives) may help improve symptoms in this population.	● Self-reported through a structured questionnaire focusing on cycle regularity, flow amount (using pad usage as a proxy), and duration.
Okoth et al., 2023^38^ Cohort study United Kingdom	N=704,743 18-40 years	CVD	Secondary amenorrhea, Polymenorrhea, Oligomenorrhea, Menorrhagia	● Irregular or abnormal menstrual cycles are linked to higher risk of cardiometabolic conditions (i.e., ischemic heart disease, cerebrovascular disease, heart failure, hypertension, T2DM). ● Menstrual cycle irregularity may serve as an early marker of future cardiometabolic dysfunction.	● Self-reported to the participant’s primary care providers during routine healthcare visits and recorded in electronic health records using diagnostic codes. ● Review of existing health records to identify exposures.
Udholm et al., 2023^40^ Cohort study Denmark	N=93,778 27-33 years	CVD	Menstrual irregularity	● Women with congenital heart disease (CHD) report slightly more menstrual irregularity than those without CHD. ● Menstrual irregularity did not affect time to pregnancy.	● Self-reported by participants during the first Danish National Birth Cohort computer-assisted telephone interview.
Wang et al., 2022^29^ Prospective cohort United States	N=80,630 14-46 years	CVD	Menorrhagia, Polymenorrhea, Oligomenorrhea	● Irregular, absent, or long menstrual cycles are associated with higher long-term cardiovascular disease (CVD) risk, especially for coronary heart disease. ● Risk persists across the life course and is largely independent of traditional risk factors, supporting menstrual history as an early marker of CVD risk.	● Self-reported menstrual cycle regularity and cycle length on validated Nurses’ Health Study II questionnaires at enrollment (1989) for ages 14–17 and 18–22 years, and updated their current menstrual characteristics in 1993 (ages 29–46 years). Cycle regularity was categorized as *very regular, regular, usually irregular, always irregular,* or *no periods*, while cycle length was reported in predefined categories.
Chakhtoura et al., 2015^8^ Retrospective cohort France	N=231 Mean age at follow-up start 41.6	CKD	Amenorrhea, Oligomenorrhea, Polymenorrhagia	● Menstrual regularity declines with dialysis (↑ amenorrhea/oligomenorrhea) and partially improves post-transplant; abnormal bleeding remains common. ● Women with CKD (dialysis/post-transplant) have high rates of menstrual and reproductive complications, requiring close gynecologic follow-up.	● Retrospective chart review.
Chang et al., 2022^7^ Exploratory mixed methods Canada	N=98 18-50 years	CKD	Secondary amenorrhea, Menorrhagia	● Menstrual disturbances are common in CKD, worsen with disease progression, and often improve after transplantation. ● Highlights need for better reproductive health discussions and multidisciplinary, person-centered care in this population.	● Self-reported on structured survey questions and in-depth participant interviews.
Jiang & Xue, 2019^6^ Retrospective Cohort China	N=58 33-48 years	CKD	Menorrhagia	● 60% of women with chronic renal failure have menstrual disorders, worsening anemia and quality of life. ● Radiofrequency endometrial ablation may be an effective minimally invasive option, but safety in this population remains uncertain.	● Pictorial Blood Loss Assessment Chart (PBAC) for self-assessment of menstrual blood loss.
Lin et al., 2016^42^ Cross-sectional China	N=184 14-44 years	CKD	Polymenorrhea, Oligomenorrhea, Secondary Amenorrhea	● Menstrual disturbances are common in ESRD, especially secondary amenorrhea (>50%), driven by HPG axis disruption and hyperprolactinemia. ● Dialysis modality and transplantation can partially restore function, but irregularities often persist due to comorbidities and treatment effects.	● Self-reported by completing a detailed questionnaire about their menstrual patterns.
Reynolds et al., 2021^44^ Qualitative United States	N=189 N=18-65 years	CKD	Oligomenorrhea, Polymenorrhea	● Regular assessment for early menopause is important due to the established elevated risk of CVD for women with glomerular disease and/or vasculitis	● Self-reported through a structured survey.
Reynolds et al., 2024^45^ Cohort United States	N=2589 18-45 years	CKD	Secondary Amenorrhea	● Within this large, nationwide Hispanic/Latino cohort, early-stage chronic kidney disease did not appear to increase the risk of infertility, loss of menses, or nonviable pregnancy loss.	● Self-reported in a health survey.
Shemies et al., 2022^41^ Cross-sectional Egypt	N=472 15-50 years	CKD	Secondary Amenorrhea, Oligomenorrhea, Polymenorrhea, Menorrhagia, Dysmenorrhea	● Menstrual disorders are highly prevalent in women with ESRD on dialysis (amenorrhea ∼33%, irregular cycles ∼37%, dysmenorrhea ∼59%). ● Sociodemographic and disease factors influence these patterns, highlighting the need for greater attention in nephrology care.	● Self-reported using a structured questionnaire administered during routine dialysis sessions; responses included details on menstrual pattern, premenstrual syndrome, and dysmenorrhea.
Sikora-Grabka et al., 2016^46^ Cross-sectional/prospective cohort Poland	N=100 Under 40 years	CKD	Secondary Amenorrhea, Oligomenorrhea, Polymenorrhea	● 80% of women with ESRD regain regular menses after kidney transplantation. ● ∼45% regain regular cycles with chronic hemodialysis.	● Detailed medical history interviews, which included questions about menstruation and fertility disorders, number of pregnancies, and the actual day of the menstrual cycle during examination. ● Cycles classified as regular (lasting 29 ± 3 days), irregular, or amenorrhea (no menstrual bleeding for more than 6 months) ● Serum Anti-Müllerian Hormone (AMH) levels.
Codner et al., 2012^47^ Systematic review Chile	N=205 20-39	T1DM	Secondary Amenorrhea, Oligomenorrhea, Polymenorrhea	● Menstrual irregularities in type 1 diabetes may reflect hypoestrogenism, potentially increasing cardiovascular risk. ● Highlights the need for comprehensive reproductive and cardiometabolic care in women with T1DM.	● Self-reported menstrual histories, clinical interviews, and hormonal measurements, with the prevalence and types of menstrual irregularities systematically documented in multiple studies.
Hassan et al., 2017^48^ Cross-sectional United States	N=89 18-50 years	T1DM	Secondary Amenorrhea. Oligomenorrhea	● Women with T1DM have higher sex hormone binding globulin and lower bioavailable estradiol, influenced by insulin dynamics and contraceptive use. ● Hormone levels vary with reproductive duration, suggesting links between insulin regulation and ovarian steroid production.	● Self-reported on structured, self-administered questionnaire.
Kim et al., 2018^49^ Secondary analysis United States	N=379 29-40 years	T1DM	Irregular menses	● Women with type 1 diabetes have higher infertility risk, with common abnormalities in ovarian markers (e.g., elevated AMH) independent of menstrual irregularity. ● These ovarian changes, rather than cycle irregularity, may partly explain reduced fecundity in this population.	● Self-reported by the women during annual, structured interviews.
Morrison et al., 2011^50^ Prospective Cohort United States	N=370 19-25 years	T2DM	Secondary Amenorrhea, Oligomenorrhea, Polymenorrhea	● Adolescents with oligomenorrhea have a much higher risk of impaired fasting glucose/T2DM in young adulthood (up to 38% vs 3% without). ● Risk increases with frequency of oligomenorrhea episodes.	● Self-reported during confidential, nurse-led annual interviews, using a clear definition (cycle length ≥42 days).
Rostami Dovom et al., 2016^10^ Prospective Cohort Iran	N=2128 18-49 years	T2DM, CVD	Oligomenorrhea	● Irregular menstrual cycles were linked to higher risk of prediabetes and diabetes, independent of BMI and family history. ● Supports metabolic screening and lifestyle interventions in women with cycle irregularities.	● Self-reported cycle patterns; classified as irregular cycles if, after two years of menarche, they reported completely irregular cycles or if 4 out of 5 cycles were longer than 35 days. ● Data collected at baseline and during follow-up as part of the Tehran Lipid and Glucose Study, using standardized questionnaires and classification criteria.
Wang et al., 2020^9^ Prospective cohort United States	N=75,546 29-46 years	T2DM	Menorrhagia, Polymenorrhea, Oligomenorrhea	● Irregular or long menstrual cycles are associated with higher risk of cardiovascular disease and type 2 diabetes. ● Risk is greater across the lifespan and may be amplified by unhealthy lifestyle factors.	● Self-reported menstrual cycle regularity and usual cycle length via Nurses’ Health Study II questionnaires. Recalled cycle characteristics at ages 14–17 years and 18–22 years at baseline (1989) and reported their current menstrual cycle characteristics in 1993 (ages 29–46 years). Menstrual regularity was categorized as *very regular, regular, usually irregular,* or *always irregular*, and cycle length was reported in predefined categories.
Saha et al., 2014^11^ Prospective cohort United States	N=121 18 and older	IBD	Polymenorrhea, Oligomenorrhea, Menorrhagia	● Menstrual disturbances (changes in cycle, flow, and pain) often occur before IBD diagnosis and may help with early detection. ● Symptoms generally stabilize after diagnosis, and appropriate therapy may improve cycle regularity and quality of life	● Self-reported via an interviewer-administered questionnaire at study entry and annually, detailing changes in cycle length, duration of flow, cycle regularity, and dysmenorrhea over the past 12 months.
Cho et al., 2022^12^ Cohort South Korea	N=72092 Under 40 years	Liver disease	Menorrhagia, Polymenorrhagia, Oligomenorrhea	● Long or irregular menstrual cycles are linked to higher risk of nonalcoholic fatty liver disease, independent of obesity and insulin resistance. ● Menstrual patterns may serve as useful markers for nonalcoholic fatty liver disease screening and prevention.	● Self-reported using a standardized, self-administered questionnaire during routine health examinations.

Legend:**AMH**: Anti-Müllerian hormone; **AUB**: Abnormal uterine bleeding; **BMI**: Body mass index; **CHD**: Coronary heart disease; **CKD**: Chronic kidney disease; **CVD**: Cardiovascular disease; **ESRD**: End-stage renal disease; **HPG**: Hypothalamic–pituitary–gonadal (axis); **HTN**: Hypertension; **IBD**: Inflammatory bowel disease; **ICD-10**: International Classification of Diseases, Tenth Revision; **MACE**: Major adverse cardiovascular events; **MI**: Myocardial infarction; **PBAC**: Pictorial Blood Loss Assessment Chart; **T1DM**: Type 1 diabetes mellitus; **T2DM**: Type 2 diabetes mellitus.

### Cardiovascular disease (CVD)

Ten studies examined the relationship between menstrual irregularities and CVD, including hypertension, coronary heart disease, myocardial infarction, atrial fibrillation, ischemic stroke, congenital heart disease, and major adverse coronary vascular events.^2–11^ Five studies derived data from retrospective chart reviews and five used cross-sectional or prospective study designs and collected self-reported data using questionnaires or computer-assisted telephone interviews. The menstrual abnormalities associated with CVD were menorrhagia, oligomenorrhea, polymenorrhea, secondary amenorrhea, dysmenorrhea, and irregular menstrual cycles. Across the majority of studies, irregular menstrual cycles (polymenorrhea and oligomenorrhea) were most frequently associated with CVD risk, followed by heavy or prolonged bleeding (menorrhagia).

Lai et al. found that polymenorrhea and oligomenorrhea were significantly associated with atrial fibrillation, hypertension, myocardial infarction, ischemic heart disease, and coronary heart disease, while dysmenorrhea was positively associated with increased risk of atrial fibrillation and cardioembolic ischemic stroke.^3^ Similarly, Okoth et al. reported that women with irregular cycles had a composite CVD incidence rate of 0.67 per 1,000 person-years compared with 0.50 among women with regular cycles, and 3.48 versus 2.79 per 1,000 person-years for hypertension, confirming higher long-term cardiometabolic risk.^9^ Collectively, these findings suggest that menstrual irregularity may serve as a potential marker of subclinical CVD risk.

Findings from congenital heart disease and perimenopausal cohorts further supported this relationship. Matsushita et al. reported excessive menstrual bleeding in 42% of women with Fontan circulation, accompanied by reduced ventricular function.^8^ Udholm et al. observed that women with congenital heart defects more frequently reported irregular periods than unaffected women.^10^ In a retrospective study among women in perimenopause, menorrhagia accounted for nearly half (48%) of abnormal uterine bleeding cases, with a high prevalence of cardiometabolic comorbidities, including hypertension (25%), diabetes (15%), and hypothyroidism (10%).^5^ Collectively, this body of evidence demonstrates a consistent association between menstrual irregularities and adverse cardiovascular outcomes across both congenital and acquired heart disease populations.

### Chronic kidney disease (CKD)

Eight of the 26 studies included in this scoping review examined menstrual irregularities in women with CKD.^12–19^ Three studies retrospectively extracted data from electronic medical records, whereas five employed prospective or cross-sectional study designs and collected self-reported data using questionnaires. Reported rates of menstrual irregularities in women with kidney failure ranged from 37% to 64.2%, and reached as high as 94%.^12,13,15,18^ The most frequently observed menstrual irregularities were amenorrhea and polymenorrhea, together affecting approximately 85% of cases.^15^ The prevalence of amenorrhea alone ranged from 32.6%^18^ to 43.1%^14^ and exceeded 50% in some cohorts.^15^

Early menopause and secondary amenorrhea were particularly common in women with glomerular disease or vasculitis.^16^ Oligomenorrhea was among the least common irregularities, reported in 25.6% of cases.^14,15^ Menorrhagia occurred in 14.8–38.9% of patients, while dysmenorrhea was reported in 58.8% of women, particularly those with autoimmune disease, hepatitis C, or mixed etiologies; it was less frequent among women who initiated dialysis after age 30.^12,18^ Metrorrhagia affected roughly 25% of women on dialysis and 30.5% of post-transplant patients.^14^ In contrast, one study found no significant association between early-stage CKD and menstrual irregularities in a Hispanic/Latino population.^17^ Findings suggest that menstrual function may serve as an indicator of renal status and overall health condition.^13^ This aligns with emerging evidence that menstrual disturbances may reflect systemic inflammation and hormonal dysregulation associated with renal impairment.

### Diabetes mellitus

Of the 26 studies included for analysis, six sources correlated menstrual irregularities and diabetes mellitus, with three focusing on T1DM^20–22^ and three on T2DM.^23–25^ One study retrospectively extracted data, one employed a cross-sectional study design with self-reported data, and the remaining four were prospective studies with self-reported data (questionnaires). Women with T1DM reported menstrual irregularities, including secondary amenorrhea, oligomenorrhea, polymenorrhea, and unspecified irregular cycles.^20–22^ Between 21% and 67% of adult women with type 1 diabetes mellitus experience menstrual irregularities, including amenorrhea, oligomenorrhea, and polymenorrhea.^20^ Secondary amenorrhea and oligomenorrhea have also been described in this population, with altered hormonal profiles characterized by lower bioavailable estradiol and higher sex hormone–binding globulin compared with controls.^21^ Irregular menses were reported in 21% of 397 women with type 1 diabetes in another study.^22^

In T2DM, menstrual irregularities were most commonly reported as secondary amenorrhea, oligomenorrhea, polymenorrhea, and menorrhagia.^23–25^ One study reported that impaired fasting glucose occurred in 38% of females aged 19–24 years who had three or more reports of oligomenorrhea during adolescence, compared with 11% among those with two reports, 6% with one report, and 3% among those with no history of oligomenorrhea.^23^ In a large cohort study, menstrual cycle irregularity was associated with a 41% increased risk of diabetes in early adulthood (ages 18–22) and a 66% increased risk in later adulthood (ages 29–46).^25^ Long menstrual cycles (≥40 days) were also associated with elevated diabetes risk, with a 37% increase at ages 18–22 and a 50% increase at ages 29–46.^25^ Similarly, women with irregular cycles had a 33% higher risk of prediabetes compared with those with regular cycles, even after adjustment for body mass index, fasting glucose, and family history.^24^ Taken together, these findings suggest that menstrual irregularities could serve as an early clinical marker of impaired glucose regulation across the diabetes spectrum.

### Inflammatory bowel disease (IBD)

One study reported on IBD and menstrual irregularities, retrospectively with self-reporting strategies that showed menstrual irregularities were common during the year preceding diagnosis.^26^ Overall, 24.8% reported changes in menstrual cycle interval, 20.7% reported changes in flow duration, and 39.6% of those who experienced menstruation reported dysmenorrhea.^26^ After diagnosis, no significant changes were observed in mean cycle length, flow or pain characteristics over time. However, cycle regularity improved, with each additional year of disease increasing the odds of a regular cycle.^26^ Women with irregular cycles also reported lower quality of life scores, suggesting that menstrual health contributes meaningfully to disease burden,^26^ highlighting the need to integrate menstrual health assessment into holistic IBD management.

### Liver disease

One study examined liver disease and menstrual irregularities with a standardized, self-administered questionnaire.^27^ In a large population-based cohort of over 72,000 premenopausal women, polymenorrhea and oligomenorrhea were significantly associated with both prevalent and incident nonalcoholic fatty liver disease (NAFLD), now termed metabolic dysfunction-associated steatotic liver disease (MASLD).^27^ At baseline, 7.1% of participants had MASLD, while 27.7% reported long or irregular menstrual cycles.^27^ Women with longer or irregular cycles were younger and more likely to have hypertension, diabetes, obesity, and elevated lipid, inflammatory, and insulin resistance markers.^27^ Compared with women with 26–30 day cycles, those with 31–39 day, ≥40 day, or irregular cycles had significantly higher prevalence ratios for MASLD, and this relationship persisted after adjustment for metabolic and lifestyle factors.^27^ Moreover, Cho et al. found that a dose-response association was observed between increasing menstrual cycle length and MASLD severity.^27^ Overall, women with irregular cycles demonstrated up to a 49% increased risk of developing MASLD during follow-up, independent of obesity and insulin resistance.^27^ These findings position menstrual irregularity as a possible early clinical marker of systemic metabolic dysfunction.

### Interventions

Six studies examining the relationship between menstrual irregularities and chronic conditions discussed interventions to manage menstrual irregularities. Interventions were categorized into surgical, pharmacologic, and medical management approaches, demonstrating varying degrees of success in improving menstrual function and related outcomes. Interventions were noted in the studies involving CVD,^6^ CKD,^12–15,19^ and IBD,^26^ while none were reported in those examining women with diabetes^20–25^ or liver disease.^27^

Surgical management was primarily reported in studies of CVD^6^ and CKD.^12–15,19^ In CVD, hysterectomy was performed for menorrhagia and proved effective in resolving heavy bleeding.^6^ However, hysterectomy was also associated with adverse metabolic outcomes, including elevated serum inflammatory markers and increased use of diabetes medications, which may predispose individuals to future coronary vascular events.^6^ In CKD, renal transplantation represented a key surgical intervention with the most significant impact on menstrual function.^13^ Following transplantation, 57.1% of women regained normal menstrual cycles, while approximately 23.1% remained amenorrheic.^13^ Restoration of renal function and a 25% reduction in serum prolactin contributed to these improvements.^15^ Self-reported rates of menstrual recovery following kidney transplantation varied across studies, with more than 80% of women resuming regular menstrual cycles in one study,^19^ compared with approximately 50% in another.^15^ Endometrial ablation was another effective, minimally invasive surgical option, resulting in amenorrhea in 89.7% of patients and high satisfaction rates, though potential effects on coagulation function were noted.^12^ Hysterectomy was reserved for those who failed or declined hormonal therapy, but the investigators noted that hysterectomy carries operative risks and causes sterility.^12^

Pharmacologic management was investigated in females with CKD and menstrual irregularities.^12,14^ Chlormadinone acetate, a synthetic progestogen, demonstrated significant efficacy in improving metrorrhagia, showing an 80% response rate at higher doses and longer durations, and a 53% response when combined with percutaneous estradiol.^14^ Additional hormonal options included oral or intrauterine progesterone and combined oral contraceptives, though supporting evidence remains limited.^12^ Tranexamic acid was also used effectively to control heavy menstrual bleeding without major complications.^12^ In women with IBD, menstrual irregularities linked to steroid therapy improved when they transitioned to steroid-sparing agents such as thiopurines or anti-TNFα therapies.^26^

Medical management strategies were described in two studies that included women with CKD.^15,19^ Lin et al. found that dialysis modification played a crucial role in normalizing serum prolactin levels and alleviating menstrual irregularity.^15^ One reason for this might be that nighttime hemodialysis (NHD) could result in a decreased peak and flattened curve of secreted prolactin.^15^ Additionally, the total dialysis time per week was significantly longer in the NHD group compared to the conventional hemodialysis (HD) group, which resulted in improved serum prolactin removal.^15^ Similarly, Sikora-Grabka et al. found that approximately 45% of women on HD reported the return of normal menses.^19^

## Discussion

### Current knowledge

This scoping review highlights the critical intersection between chronic health conditions and menstrual health, identifying menstrual irregularities as a common yet frequently overlooked consequence of chronic disease in women. Across cardiovascular, renal, metabolic, inflammatory, and hepatic conditions, women consistently experienced menstrual disturbances. The underlying physiology has been attributed to hormonal dysregulation involving the HPA axis and hypothalamic allostasis.^30–32^ The long-term metabolic consequences associated with HPA axis dysregulation may contribute to important downstream health effects.^30–32^ Despite growing recognition of menstrual health as an underrecognized aspect of chronic condition management and an important indicator of systemic well-being, it remains poorly integrated into chronic condition care and treatment planning. Across studies, irregular cycles, amenorrhea, and menorrhagia were the most frequently reported menstrual disturbances, commonly linked to chronic conditions through hormonal, metabolic, and inflammatory mechanisms.^3,13,15,23,25,27^ However, standardized approaches to menstrual assessment and management were notably absent, revealing a critical gap in women’s health research and clinical practice with potential long term health consequences.

Menstrual irregularities were prevalent across all chronic condition categories included in the review and have consequences extending beyond reproductive health. Unmanaged bleeding or amenorrhea can contribute to chronic anemia, fatigue, infertility, and reduced quality of life.^18,25,26^ Furthermore, the absence of estrogenic protection associated with irregular or absent menstrual cycles has been linked to heightened cardiometabolic risk, including hypertension and myocardial infarction.^3,9,35^ Mattina et al. describe how declining ovarian function and reduced estrogen, common contributors to menstrual dysfunction, promote endothelial dysfunction, systemic inflammation, and immune dysregulation, collectively increasing cardiovascular disease risk.^52–55^ These findings reinforce the central role of estrogen in maintaining vascular integrity and suggest that menstrual irregularities serve as reproductive symptoms but also as biomarkers of hypothalamic allostasis. Neglecting menstrual health during routine clinical care may therefore contribute to delayed diagnosis, inadequate disease monitoring, and worsening systemic complications.

### Management of menstrual irregularities

Management strategies reported across the literature were fragmented and inconsistently described. Most interventions focused primarily on symptom control rather than addressing the underlying systemic mechanisms responsible for menstrual dysfunction. Hormonal and pharmacologic therapies demonstrated improvements in menstrual symptoms among women with CKD but were rarely evaluated in other chronic conditions, leaving uncertainty regarding their broader effectiveness and potential cardiovascular or metabolic risks.^12,14,15^ Surgical interventions, including hysterectomy and endometrial ablation, effectively reduced abnormal uterine bleeding but did not address the endocrine or inflammatory mechanisms driving menstrual dysfunction and may worsen metabolic complications in some women.^6,12^

Restoration of renal function following kidney transplantation was associated with normalization of prolactin levels and resumption of regular menstrual cycles in approximately 50% to 80% of women, illustrating that treatment of the underlying systemic disease can restore reproductive function.^14–19^ However, persistent amenorrhea following transplantation demonstrates that disease-specific treatment alone may not fully resolve menstrual dysfunction and highlights the need for comprehensive multidisciplinary care that simultaneously addresses both chronic disease and reproductive health.^13^ While these findings suggest that treating the underlying disease may restore reproductive function, the reliance on self-reported menstrual outcomes and the absence of standardized prospective menstrual monitoring limit comparisons across studies.

### Knowledge gaps and future research

Despite increasing recognition of the relationship between chronic conditions and menstrual health, substantial gaps remain in the evidence base, particularly regarding prevalence, causality, and the underlying physiological mechanisms. Standardized approaches to menstrual assessment and management were largely absent across the included studies, making comparisons between chronic conditions difficult and limiting the development of evidence-based recommendations. The majority of available studies were observational, cross-sectional, retrospective, and conducted in high-income countries, limiting both causal inference and generalizability. Few investigations considered the influence of social determinants of health, cultural stigma surrounding menstruation, or access to menstrual products on symptom reporting and management.^16^˒^26^ Likewise, little evidence exists regarding the long-term cardiovascular, metabolic, and reproductive consequences of chronic condition-related menstrual dysfunction. The allostatic load hypothesis provides a plausible mechanistic framework whereby chronic physiological stress and sustained activation of the hypothalamic-pituitary-adrenal (HPA) axis disrupt hypothalamic-pituitary-gonadal axis function, ultimately impairing ovulation and menstrual cyclicity.^30–32^ Future longitudinal and interventional studies are needed to investigate this proposed pathway, clarify disease-specific mechanisms, and determine whether reducing allostatic load or improving chronic disease control can mitigate menstrual dysfunction and its downstream health consequences.

An important finding of this review is the assessment of menstrual irregularities. Many studies relied on retrospectively collected self-reported menstrual histories or questionnaires, while others used the prospective assessment of menstrual cycle characteristics. Although self-reported menstrual characteristics are widely used in epidemiologic research, retrospective recall may underestimate or misclassify menstrual abnormalities, particularly among women managing complex chronic illnesses. Future research should prioritize standardized prospective menstrual assessment using validated questionnaires, electronic menstrual diaries, or digital health applications, with implementation tailored to available resources. For example, questionnaires embedded within electronic medical records or mobile health applications may improve accessibility in high-income settings, whereas paper-based questionnaires or telephone interviews may be more feasible in resource-limited settings.

### Clinical practice implications

The findings of this review support integrating standardized menstrual health assessment into routine chronic disease care and recognizing menstrual health as an essential component of chronic condition management rather than a separate aspect of reproductive care. Regular menstrual screening offers a practical, economical, and non-invasive method for identifying early physiological changes associated with disease progression while providing insight into treatment effectiveness and overall health status. Primary care providers and specialists are ideally positioned to incorporate routine menstrual history into chronic disease assessments, recognizing menstrual changes as clinically meaningful indicators of systemic health rather than isolated reproductive concerns.^13^˒^16^ Routine menstrual inquiry may reveal early evidence of endocrine dysfunction, treatment-related adverse effects, or worsening systemic disease that might otherwise remain undetected. Recognizing the menstrual cycle as a “sixth vital sign” provides clinicians with an accessible biomarker of systemic health that can facilitate earlier diagnosis, improve monitoring of chronic disease progression, and guide individualized treatment.^54^ Embedding standardized menstrual assessment and cycle documentation within electronic health records and chronic disease management tools, together with the development of evidence-based clinical guidelines, could improve interdisciplinary communication, continuity of care, earlier identification and referral, and ultimately promote more equitable, comprehensive, and patient-centred care for women who menstruate across the lifespan.

### Future Research

Future research should focus on identifying the mechanisms linking chronic conditions and menstrual irregularities, evaluating their long-term health consequences, and developing evidence-based, multidisciplinary models of care that integrate menstrual health into chronic disease prevention and management. By advancing this knowledge, healthcare providers, researchers, and policymakers can work toward a more equitable, compassionate, and sex- and gender-informed healthcare system in which menstrual health is recognized as a fundamental indicator of overall health and women’s symptoms are consistently acknowledged, investigated, and addressed.

### Limitations

This review has several limitations. Only English-language studies were included, potentially excluding relevant research from other regions and limiting global representation. Most studies originate from high-income countries, indicating a potential gap in representation from low- and middle-income countries. Due to the limited number of studies on this topic, some included studies involved participants outside the target age range; these were retained if they provided age-stratified data or if the majority of participants were aged 18 - 45 years. Data extraction was completed by a single reviewer due to time and resource limitations, which may have introduced selection bias despite accuracy checks. Finally, heterogeneity in study design and definitions of menstrual irregularities limited comparability across studies and restricted causal interpretation. Despite these limitations, this review provides a solid foundation for future research and clinical inquiries in menstrual health and chronic condition management.

## Conclusion

This review identified substantial inconsistencies in the recognition, assessment, reporting, and management of menstrual irregularities, with most interventions focusing on symptom management rather than addressing the underlying physiological mechanisms. Consequently, many women may remain vulnerable to the long-term effects of persistent hypoestrogenemia, including increased risks of cardiovascular disease,^37,38^ osteoporosis,^40^ and neurological complications.^41^ Recognizing menstrual health as an integral component of chronic disease management, rather than as an isolated aspect of reproductive health, may provide an important opportunity to improve patient care. Routine menstrual assessment can serve as a valuable clinical indicator of systemic health, facilitating earlier identification of physiological changes, informing treatment decisions, and supporting more comprehensive, patient-centred care. Integrating standardized menstrual screening into chronic disease management could improve health outcomes while addressing an important gap in women’s healthcare. The authors propose implementing a standardized menstrual health questionnaire within specialty clinics to facilitate consistent assessment and earlier recognition of menstrual disturbances, with the goal of supporting timely intervention.

## Supplemental material

Supplemental material - The overlooked connection: Managing menstrual irregularities in chronic conditions - A scoping review Supplemental material for The overlooked connection: Managing menstrual irregularities in chronic conditions - A scoping review by Jennifer L. Dueck, Hilary Hwu, Simin Popat, Alena K. Thompson, Nicole L. Tegg, Colleen M. Norris in Women's Health

## Consent for publication

Consent for publication was not required for this study as it used published data only.

## Data Availability

No new data were generated during this study. All analyzed data were obtained from previously published peer-reviewed articles and are fully cited and included within this manuscript and its supplementary files.*
