# Supplementary material for: The overlooked connection: Managing menstrual irregularities in chronic conditions - A scoping review
Source: Womens Health (Lond). 2026 Sep 29;22:17455057261493736. doi: 10.1177/17455057261493736 (PMC13735775; doi:10.1177/17455057261493736)
Supplement: Supplementary file 1 [file sj-pdf-1-whe-10.1177_17455057261493736.pdf]

Appendix A: Search Strategy

MEDLINE (1946-present via Ovid) - May 27, 2025
Records retrieved: 537

#	Searches
1	((menstrua* adj3 (disrupt* or disturb* or stop* or absent or absence or irregular*)) or Amenorrh?ea or oligomenorrh?ea or polymenorrh?ea or menorrhagia or hypomenorrh?ea).mp.
2	(chronic* adj3 (disease* or ill* or condition* or disorder*)).mp.
3	(CVD or ((cardio* or heart or cardiac) adj1 disease*) or hypertension or "high blood pressure" or "coronary artery disease" or stroke or "cardiac valve disease" or cardiomyopathy).mp.
4	(("type 1" adj2 diabet*) or "insulin dependent diabet*" or T1D or TID).mp.
5	("inflammatory bowel disease*" or IBD).mp.
6	((chronic adj2 (kidney or renal)) or CKD).mp.
7	(Chronic adj2 (liver or hepatic or cirrhosis)).mp.
8	2 or 3 or 4 or 5 or 6 or 7
9	adult*.mp.
10	1 and 8 and 9
11	limit 10 to (english language and yr="2010 -Current")

MEDLINE T2DM - July 5, 2025

#	Searches
1	((menstrua* adj3 (disrupt* or disturb* or stop* or absent or absence or irregular*)) or Amenorrh?ea or oligomenorrh?ea or polymenorrh?ea or menorrhagia or hypomenorrh?ea).mp.
2	(chronic* adj3 (disease* or ill* or condition* or disorder*)).mp.
3	(("type 2" adj2 diabet*) or "non-insulin dependent diabet*" or T2D or “adult onset diabetes).mp.
4	2 OR 3

5	adult*.mp.
6	1 AND 4 AND 5
7	limit 6 to (english language and yr="2010 -Current")

Embase (Ovid) - May 27, 2025
Records retrieved: 4197

#	Searches
1	((menstrua* adj3 (disrupt* or disturb* or stop* or absent or absence or irregular*)) or Amenorrh?ea or oligomenorrh?ea or polymenorrh?ea or menorrhagia or hypomenorrh?ea).mp.
2	(chronic* adj3 (disease* or ill* or condition* or disorder*)).mp.
3	(CVD or ((cardio* or heart or cardiac) adj1 disease*) or hypertension or "high blood pressure" or "coronary artery disease" or stroke or "cardiac valve disease" or cardiomyopathy).mp.
4	(("type 1" adj2 diabet*) or "insulin dependent diabet*" or T1D or TID).mp.
5	("inflammatory bowel disease*" or IBD).mp.
6	((chronic adj2 (kidney or renal)) or CKD).mp.
7	(Chronic adj2 (liver or hepatic or cirrhosis)).mp.
8	2 or 3 or 4 or 5 or 6 or 7
9	adult*.mp.
10	1 and 8 and 9
11	limit 10 to (english language and yr="2010 -Current")

Embase T2DM - July 5, 2025

#	Searches
1	((menstrua* adj3 (disrupt* or disturb* or stop* or absent or absence or irregular*)) or Amenorrh?ea or oligomenorrh?ea or polymenorrh?ea or menorrhagia or hypomenorrh?ea).mp.
2	(chronic* adj3 (disease* or ill* or condition* or disorder*)).mp.
3	(("type 2" adj2 diabet*) or "non-insulin dependent diabet*" or T2D or “adult onset

	diabetes).mp.
4	2 OR 3
5	adult*.mp.
6	1 AND 4 AND 5
7	limit 6 to (english language and yr="2010 -Current")

CINAHL Plus with Full Text - May 27, 2025
Records retrieved: 160

#	Searches
S1	((menstrua* N3 (disrupt* or disturb* or stop* or absent or absence or irregular*)) or Amenorrh#ea or oligomenorrh#ea or polymenorrh#ea or menorrhagia or hypomenorrh#ea)
S2	(chronic* N3 (disease* or ill* or condition* or disorder*))
S3	(CVD or ((cardio* or heart or cardiac) N3 disease*) or hypertension or "high blood pressure" or "coronary artery disease" or stroke or "cardiac valve disease" or cardiomyopathy)
S4	(("type 1" N2 diabet*) or "insulin dependent diabet*" or T1D or TID)
S5	("inflammatory bowel disease*" or IBD)
S6	((chronic N2 (kidney or renal)) or CKD)
S7	(Chronic N2 (liver or hepatic or cirrhosis))
S8	S2 OR S3 OR S4 OR S5 OR S6 OR S7
S9	adult*
S10	S1 AND S8 AND S9
S11	S1 AND S8 AND S9 Limiters - English Language
S12	S1 AND S8 AND S9 Limiters - English Language; Publication Date: 20100101-20251231

CINAHL T2DM - July 5, 2025

#	Searches

S1	((menstrua* N3 (disrupt* or disturb* or stop* or absent or absence or irregular*)) or Amenorrh#ea or oligomenorrh#ea or polymenorrh#ea or menorrhagia or hypomenorrh#ea)
S2	(chronic* N3 (disease* or ill* or condition* or disorder*))
S3	(("type 2" N2 diabet*) or "non-insulin dependent diabet*" or T2D or “adult onset diabetes)
S4	S2 OR S3
S5	adult*
S6	S1 AND S4 AND S5
S7	Limiters - English Language; Publication Date: 20100101-20261231
S8	S6 AND S7
S9	S6 AND S7 Narrow by Language: -english

Scopus - May 13, 2025
Records retrieved: 1091

TITLE-ABS-KEY ((menstrua* W/3 (disrupt* OR disturb* OR stop* OR absent OR absence OR irregular*)) OR amenorrh?ea OR oligomenorrh?ea OR polymenorrh?ea OR menorrhagia OR hypomenorrh?ea) OR TITLE-ABS-KEY (chronic* W/3 (disease* OR ill* OR condition* OR disorder*))
OR TITLE-ABS-KEY (CVD OR (cardio* OR heart OR cardiac) W/1 disease*)
OR TITLE-ABS-KEY (hypertension OR "high blood pressure" OR "coronary artery disease" OR stroke OR "cardiac valve disease" OR cardiomyopathy)
OR TITLE-ABS-KEY (("type 1" W/2 diabet*) OR "insulin dependent diabet*" OR T1D OR TID)
OR TITLE-ABS-KEY ("inflammatory bowel disease*" OR IBD)
OR TITLE-ABS-KEY ((chronic W/2 (kidney OR renal)) OR CKD)
OR TITLE-ABS-KEY ((chronic W/2 (liver OR hepatic OR cirrhosis)))
Adult*

Scopus T2DM - July 5, 2025

TITLE-ABS-KEY (menstrua* W/3 (disrupt* OR disturb* OR stop* OR absent OR absence OR irregular*)) OR amenorrh?ea OR oligomenorrh?ea OR polymenorrh?ea OR menorrhagia OR hypomenorrh?ea) AND TITLE-ABS-KEY chronic* W/3 (disease* OR ill* OR condition* OR disorder*)
OR TITLE-ABS-KEY ("type 2" W/2 diabet*) OR "non-insulin dependent diabet*" OR T2D OR adult onset diabetes)

Web of Science - June 3, 2025 ("All databases" and "core collection")
Records retrieved: 541

#	Searches
1	TS=(((menstrua* NEAR/3 (disrupt* or disturb* or stop* or absent or absence or irregular*)) or Amenorrh?ea or oligomenorrh?ea or polymenorrh?ea or menorrhagia or hypomenorrh?ea)) and Preprint Citation Index (Exclude - Database)
2	TS=((chronic* NEAR/3 (disease* or ill* or condition* or disorder*))) and Preprint Citation Index (Exclude - Database)
3	TS=((CVD or ((cardio* or heart or cardiac) NEAR/1 disease*) or hypertension or "high blood pressure" or "coronary artery disease" or stroke or "cardiac valve disease" or cardiomyopathy) and Preprint Citation Index (Exclude - Database)
4	TS=((("type 1" NEAR/2 diabet*) or "insulin dependent diabet*" or T1D or TID)) and Preprint Citation Index (Exclude - Database)
5	TS=(("inflammatory bowel disease*" or IBD)) and Preprint Citation Index (Exclude - Database)
6	TS=(((chronic NEAR/2 (kidney or renal)) or CKD)) and Preprint Citation Index (Exclude - Database)
7	TS=((Chronic NEAR/2 (liver or hepatic or cirrhosis))) and Preprint Citation Index (Exclude - Database)
8	#2 OR #3 OR #4 OR #5 OR #6 OR #7 and Preprint Citation Index (Exclude - Database)
9	TS=(adult*) and Preprint Citation Index (Exclude - Database)
10	#1 AND #8 AND #9 and Preprint Citation Index (Exclude - Database)
11	#1 AND #8 AND #9 and Preprint Citation Index (Exclude - Database) and Article or Review Article (Document Types)
12	#1 AND #8 AND #9 and Preprint Citation Index (Exclude - Database) and Article or Review Article (Document Types) and 2025 or 2024 or 2023 or 2022 or 2021 or 2020 or 2019 or 2018 or 2017 or 2016 or 2015 or 2014 or 2013 or 2012 or 2011 or 2010 (Publication Years)
13	#1 AND #8 AND #9 and Preprint Citation Index (Exclude - Database) and Article or Review Article (Document Types) and 2025 or 2024 or 2023 or 2022 or 2021 or 2020 or 2019 or 2018 or 2017 or 2016 or 2015 or 2014 or 2013 or 2012 or 2011 or 2010 (Publication Years) and English (Languages)

Web of Science T2DM - July 5, 2025

#	Searches
1	TS=(((menstrua* NEAR/3 (disrupt* or disturb* or stop* or absent or absence or irregular*)) or Amenorrh?ea or oligomenorrh?ea or polymenorrh?ea or menorrhagia or hypomenorrh?ea)) and Preprint Citation Index (Exclude - Database)
2	TS=((chronic* NEAR/3 (disease* or ill* or condition* or disorder*))) and Preprint Citation Index (Exclude - Database)
3	TS=(((type2" NEAR/2 diabet*) or "non-insulin dependent diabet*" or T2D or adult onset diabetes)) and Preprint Citation Index (Exclude - Database)
4	#2 OR #3 and Preprint Citation Index (Exclude - Database)
5	TS=(adult*) and Preprint Citation Index (Exclude - Database)
6	#1 AND #4 AND #5 and Preprint Citation Index (Exclude - Database)
7	#1 AND #4 AND #5 and Preprint Citation Index (Exclude - Database)
8	#1 AND #4 AND #5 and Preprint Citation Index (Exclude - Database) and 2025 or 2024 or 2023 or 2022 or 2021 or 2020 or 2019 or 2018 or 2017 or 2016 or 2015 or 2014 or 2013 or 2012 or 2011 or 2010 (Publication Years)
9	#1 AND #4 AND #5 and Preprint Citation Index (Exclude - Database) and 2025 or 2024 or 2023 or 2022 or 2021 or 2020 or 2019 or 2018 or 2017 or 2016 or 2015 or 2014 or 2013 or 2012 or 2011 or 2010 (Publication Years) and English (Languages)

Appendix B
Grey Literature Search

Source searched	Date searched	Search description	Results
CIHI - Canadian Institute for Health Information	Sept 5, 2025	Menstrual irregularities AND<br>• Chronic conditions<br>• Cardiovascular disease<br>• Diabetes<br>• Inflammatory bowel disease<br>• Kidney or renal disease<br>• Liver or hepatic or	0 relevant

		cirrhosis disease<br>• Chronic disease<br><br>Menstrual irregularities	
Health Canada	Sept 6, 2025	Menstrual irregularities and chronic disease<br><br>menstrual irregularit* and cardiovascular disease<br><br>menstrual irregularit* and chronic diseas*	0 relevant
WHO IRIS (https://iris.who.int)<br>And WHO (https://www.who.int/data/gho)	Sept 5, 2025	"Menstrual irregularities" and "chronic disease"<br><br>"menstrual irregularit*" and "cardiovascular disease"<br><br>"menstrual irregularit*" and "chronic diseas*"	16 (2010-2019)<br>8 (2020-2025)<br>1 relevant uploaded
WHO via GOOGLE site:who.int "menstrual irregularities" and "chronic diseases"	Sept 6, 2025	"Menstrual irregularities" and "chronic disease"<br><br>"menstrual irregularit*" and "cardiovascular disease"<br><br>"menstrual irregularit*" and "chronic diseas*"	30 sources found<br>0 relevant

Appendix C

Preferred Reporting Items for Systematic reviews and Meta-Analyses extension for Scoping Reviews (PRISMA-ScR) Checklist

SECTION	ITEM	PRISMA-ScR CHECKLIST ITEM	REPORTED ON PAGE #
**TITLE**			
**Title**	**1**	**Identify the report as a scoping review.**	**1**
**ABSTRACT**			
**Structured summary**	**2**	**Provide a structured summary that includes (as applicable): background, objectives, eligibility criteria, sources of**	**2**

		**evidence, charting methods, results, and conclusions that relate to the review questions and objectives.**	
**INTRODUCTION**			
**Rationale**	**3**	**Describe the rationale for the review in the context of what is already known. Explain why the review questions/objectives lend themselves to a scoping review approach.**	**3**
**Objectives**	**4**	**Provide an explicit statement of the questions and objectives being addressed with reference to their key elements (e.g., population or participants, concepts, and context) or other relevant key elements used to conceptualize the review questions and/or objectives.**	**3**
**METHODS**			
**Protocol and registration**	**5**	**Indicate whether a review protocol exists; state if and where it can be accessed (e.g., a Web address); and if available, provide registration information, including the registration number.**	**3**
**Eligibility criteria**	**6**	**Specify characteristics of the sources of evidence used as eligibility criteria (e.g., years considered, language, and publication status), and provide a rationale.**	**4**
**Information sources***	**7**	**Describe all information sources in the search (e.g., databases with dates of coverage and contact with authors to identify additional sources), as well as the date the most recent search was executed.**	**5**
**Search**	**8**	**Present the full electronic search strategy for at least 1 database, including any limits used, such that it could be repeated.**	**5**
**Selection of sources of evidence†**	**9**	**State the process for selecting sources of evidence (i.e., screening and eligibility) included in the scoping review.**	**5**
**Data charting process‡**	**10**	**Describe the methods of charting data from the included sources of evidence (e.g., calibrated forms or forms that have been tested by the team before their use, and whether data charting was done independently or in duplicate) and any processes for obtaining and confirming data from investigators.**	**6**

**Data items**	**11**	**List and define all variables for which data were sought and any assumptions and simplifications made.**	**6**
**Critical appraisal of individual sources of evidence§**	**12**	**If done, provide a rationale for conducting a critical appraisal of included sources of evidence; describe the methods used and how this information was used in any data synthesis (if appropriate).**	**N/A**
**Synthesis of results**	**13**	**Describe the methods of handling and summarizing the data that were charted.**	**6**
**RESULTS**			
**Selection of sources of evidence**	**14**	**Give numbers of sources of evidence screened, assessed for eligibility, and included in the review, with reasons for exclusions at each stage, ideally using a flow diagram.**	**Figure 1**
**Characteristics of sources of evidence**	**15**	**For each source of evidence, present characteristics for which data were charted and provide the citations.**	**Table 1, pages 9-11**
**Critical appraisal within sources of evidence**	**16**	**If done, present data on critical appraisal of included sources of evidence (see item 12).**	**N/A**
**Results of individual sources of evidence**	**17**	**For each included source of evidence, present the relevant data that were charted that relate to the review questions and objectives.**	**Table 1, pages 9-11**
**Synthesis of results**	**18**	**Summarize and/or present the charting results as they relate to the review questions and objectives.**	**9-11**
**DISCUSSION**			
**Summary of evidence**	**19**	**Summarize the main results (including an overview of concepts, themes, and types of evidence available), link to the review questions and objectives, and consider the relevance to key groups.**	**9-11**
**Limitations**	**20**	**Discuss the limitations of the scoping review process.**	**11**
**Conclusions**	**21**	**Provide a general interpretation of the results with respect to the review questions and objectives, as well as potential implications and/or next steps.**	**11-12**
**FUNDING**			
**Funding**	**22**	**Describe sources of funding for the included sources of evidence, as well as sources of funding for the scoping review. Describe the role of the funders of the**	**12**

		**scoping review.**	

JBI = Joanna Briggs Institute; PRISMA-ScR = Preferred Reporting Items for Systematic reviews and Meta-Analyses extension for Scoping Reviews.
* Where *sources of evidence* (see second footnote) are compiled from, such as bibliographic databases, social media platforms, and Web sites.
† A more inclusive/heterogeneous term used to account for the different types of evidence or data sources (e.g., quantitative and/or qualitative research, expert opinion, and policy documents) that may be eligible in a scoping review as opposed to only studies. This is not to be confused with *information sources* (see first footnote).
‡ The frameworks by Arksey and O'Malley (6) and Levac and colleagues (7) and the JBI guidance (4, 5) refer to the process of data extraction in a scoping review as data charting.
§ The process of systematically examining research evidence to assess its validity, results, and relevance before using it to inform a decision. This term is used for items 12 and 19 instead of "risk of bias" (which is more applicable to systematic reviews of interventions) to include and acknowledge the various sources of evidence that may be used in a scoping review (e.g., quantitative and/or qualitative research, expert opinion, and policy document).

From: Tricco AC, Lillie E, Zarin W, O'Brien KK, Colquhoun H, Levac D, et al. PRISMA Extension for Scoping Reviews (PRISMAScR): Checklist and Explanation. Ann Intern Med. 2018;169:467–473. doi: 10.7326/M18-08a50.

Appendix D: Covidence Data Extraction Table

Data extraction category	Variables extracted
Study identification	Title; First author; DOI; Year of publication; Country where the study was conducted (United States, United Kingdom, Canada, Australia, Other)
Study characteristics	Aim of the study; Study design (randomized controlled trial, non-randomized experimental study, cohort study, cross-sectional study, case-control study, systematic review, qualitative research, prevalence study, case series, case report, diagnostic test accuracy study, clinical prediction rule, economic evaluation, text and opinion, other)
Participant characteristics	Total number of participants; Inclusion criteria; Exclusion criteria; Age of participants

Clinical characteristics	Type of chronic illness (main outcome); Type of menstrual irregularity
Outcomes	Intervention for menstrual irregularities (yes/no; if yes, specify); Results/key findings; Method used to report menstrual irregularities (added during revision stage)
